# Association Between Erectile Dysfunction and Lower Urinary Tract Symptoms in a North Indian Tertiary Care Hospital

**DOI:** 10.7759/cureus.85352

**Published:** 2025-06-04

**Authors:** Rishikant Gyani, Apul Goel, Satyanarayan Sankhwar, Bhupendra P Singh, Manoj Kumar, Vivek Kumar Singh

**Affiliations:** 1 Urology, King George's Medical University, Lucknow, IND; 2 Urology, Institute of Medical Sciences, Varanasi, IND

**Keywords:** benign prostatic hyperplasia, erectile dysfunction, international index of erectile function, international prostate symptom score, lower urinary tract symptoms

## Abstract

Introduction

This study aimed to investigate the relationship between erectile dysfunction (ED) and lower urinary tract symptoms (LUTS) in a North Indian population, as well as to assess the influence of age, serum prostate-specific antigen (PSA), and testosterone levels on this association.

Materials and methods

A cross-sectional study was conducted at King George’s Medical University, Lucknow, India, involving 183 men aged ≥50 years presenting with LUTS or benign prostatic hyperplasia (BPH). The severity of ED was assessed using the International Index of Erectile Function-15 (IIEF-15), while LUTS severity was measured using the International Prostate Symptom Score (IPSS). Serum PSA and testosterone levels were evaluated. Statistical analysis was performed using IBM SPSS Statistics for Windows, Version 26.0 (Released 2018; IBM Corp., Armonk, NY, United States), with a significance level set at p < 0.05.

Results

The patients' mean ± SD age was 63.13 ± 8.98 years. The severity of LUTS was classified as mild in 67 patients (36.6%), moderate in 62 (33.8%), and severe in 54 (29.5%). ED was categorized as mild in 92 patients (50.3%), moderate in 73 (39.9%), and severe in 18 (9.8%). A significant correlation was found between ED severity and increasing age (p < 0.001), as well as with higher IPSS (p < 0.05). Additionally, ED was significantly associated with comorbidities, including hypertension (HTN) and diabetes mellitus (DM) (p < 0.05). Furthermore, IIEF-15 scores showed a significant correlation with testosterone levels (r = 0.679, p < 0.001). No significant correlation was found between ED severity and serum PSA levels.

Conclusions

This study found a significant association between ED and the severity of LUTS in men with BPH. The data revealed that patients over 79 years of age tended to have lower testosterone levels, and the presence of comorbidities such as HTN and DM emerged as major risk factors for ED. These findings highlight the significance of adopting a comprehensive, multifactorial approach to assess and manage ED in patients with LUTS.

## Introduction

Lower urinary tract symptoms (LUTS) are highly prevalent among male adults, affecting approximately 77.9% of individuals aged 20-29 years and increasing to 82.5% among those aged 40-49 years, with storage symptoms being the most commonly reported subtype. Based on the International Prostate Symptom Score (IPSS), LUTS are categorized into storage, voiding, and postvoiding symptoms. Storage symptoms, including urinary frequency, urgency, and nocturia, were the most prevalent, reported by 77.9% of individuals aged 20-29 years and 79.1% of those aged 40-49. Voiding symptoms, such as weak urinary stream, intermittency, and straining, were reported by 28.6% and 32.5% in the respective age groups. Postvoiding symptoms, including a sensation of incomplete emptying and post-micturition dribbling, were less common, observed in 17.4% and 18.5% of participants in the same age groups mentioned. These symptoms are frequently associated with benign prostatic hyperplasia (BPH), a condition characterized by the non-cancerous enlargement of the prostate gland that affects a substantial proportion of males as they age [1,2]. In addition to LUTS, erectile dysfunction (ED) is another prevalent issue, characterized by the persistent inability to achieve or maintain an erection sufficient for satisfactory sexual performance. The coexistence of LUTS and ED is well-documented, with both conditions often exacerbated by advancing age, underlying medical conditions, and hormonal imbalances. There is a linear increase in the prevalence of ED with age, with rates ranging from roughly 2% to 9% for men younger than 40 years and 18% to 86% for those older than 80 [3,4]. In this study, no Indian data were available to fully understand the national context of these global trends.

The pathophysiological mechanisms linking LUTS and ED are complex and multifactorial. Age-related changes in the prostate, coupled with alterations in hormonal levels such as testosterone, have been implicated in the development and progression of both conditions [5]. Testosterone is crucial for maintaining normal erectile function, and its deficiency is known to contribute to ED [6]. Additionally, increased prostate-specific antigen (PSA) levels, often associated with BPH, may provide insights into the severity of prostate enlargement and its impact on sexual health [7,8]. Despite the established connections between these variables, comprehensive understanding remains elusive, particularly concerning their interactions and the specific role of testosterone and PSA levels in ED severity [6,9]. Thus, this study aims to investigate the association between LUTS severity and ED in a cohort of men aged ≥50 years from North India, focusing on the interplay between age, testosterone levels, PSA levels, and comorbid conditions. By elucidating these relationships, the study seeks to contribute valuable insights into the management of LUTS and ED and therapeutic outcomes for the affected individuals.

## Materials and methods

Study design

This prospective cross-sectional study was conducted in the Department of Urology at King George's Medical University, Lucknow, India, between 2019 and 2020. This study was approved by the Institutional Ethics Committee, and informed consent was obtained from all participants before enrolment. A total of 183 male patients presenting with LUTS associated with BPH were included. The sample size was determined based on previously reported prevalence data [10]. LUTS severity was assessed using the IPSS, a validated seven-item questionnaire that categorizes symptom severity as mild (0-7), moderate (8-19), and severe (20-35) based on their total score. ED was evaluated using the five-item version of the IIEF-15, with severity classified as severe (1-7), moderate (8-11), mild-to-moderate (12-16), mild (17-21), and no ED (22-25). Exclusion criteria included a history of transurethral resection of the prostate (TURP), urinary retention/neurogenic bladder, pelvic surgery, prostate cancer, or current treatment for erectile dysfunction. These criteria were applied to ensure a focused study population and to minimize confounding factors that could affect the validity of the findings [5,11].

Assessment of LUTS and ED

LUTS and ED were evaluated using validated, self-administered questionnaires as part of a comprehensive health investigation. LUTS severity was measured using the IPSS, a standardized tool comprising seven questions evaluating urinary symptoms and their impact on quality of life. Each question is scored on a scale from 0 to 5, resulting in a total score ranging from 0 to 35. Based on the total score, LUTS severity was categorized as absent (score of 0), mild (1-7), moderate (8-19), or severe (20-35) [12]. ED was assessed using the IIEF-15, a comprehensive tool assessing multiple domains of sexual function. Each question is scored from 1 to 5, with total scores ranging from 5 to 25. Based on these scores, ED was categorized as no ED (22-25), mild (17-21), moderate (8-16), or severe (<7) [13]. All enrolled participants completed both the IPSS and IIEF-15 questionnaires. Scoring and classification were conducted according to standardized criteria.

Laboratory measurements

Serum testosterone levels and PSA levels were measured to assess hormonal status and prostate health. Serum testosterone levels were quantified using a chemiluminescent immunoassay, with normal reference ranges defined according to established laboratory standards [14]. PSA levels were measured using an enzyme-linked immunosorbent assay, with a serum PSA value ≤4.0 ng/mL considered within the normal range, based on standard clinical guidelines [15].

Statistical analysis

Data were presented as mean ± SD, and descriptive statistics were used to summarize participant demographics and the severity of LUTS and ED. Associations between LUTS severity and ED were assessed using the Kruskal-Wallis one-way analysis of variance (ANOVA), an appropriate non-parametric method for ordinal data. Chi-square tests and Spearman’s rho correlation coefficients were calculated to evaluate the relationships between testosterone levels, PSA levels, and ED severity. A p < 0.05 was considered statistically significant for all analyses. Statistical analyses were performed using IBM SPSS Statistics for Windows, Version 26.0 (Released 2018; IBM Corp., Armonk, NY, US) [16].

## Results

Patients’ characteristics

The study included n = 183 male patients, with a mean age of 63.13 ± 8.98 years (range: 50 to >79 years). The majority of patients (38.25%) belonged to the 60-69 age group, followed by the 50-59 age group (34.4%). BPH was identified as the leading cause of LUTS in this study. LUTS severity, as assessed by the IPSS, was categorized as mild in 36.6% of patients, moderate in 33.8%, and severe in 29.5%. ED, evaluated using the IIEF-15, was classified as mild in 50.3% of patients, moderate in 39.9%, and severe in 9.8% (Table 1).

**Table 1:** Patients' characteristics LUTS: lower urinary tract symptoms, ED: erectile dysfunction, IPSS: International Prostate Symptom Score, IIEF-15: International Index of Erectile Function-15.

Parameter	Value
Total number of patients	183
Mean ± SD age (years)	63.13 ± 8.98
Age range (years)	50-79
Age distribution	
50-59	63 (34.42)
60-69	70 (38.25)
70-79	39 (21.31)
>79	11 (6.01)
LUTS severity (IPSS)	
Mild	67 (36.6%)
Moderate	62 (33.8%)
Severe	54 (29.5%)
ED severity (IIEF-15 score)	
Mild	92 (50.3%)
Moderate	73 (39.9%)
Severe	18 (9.8%)

Associations among degrees of ED with age, duration of symptoms, LUTS severity, PSA, and testosterone levels

Significant associations were observed between ED severity and patient age, duration of LUTS (including urinary frequency, urgency, nocturia, weak stream, and other related complaints), IPSS, PSA, and testosterone levels. Mild ED was most commonly observed in men aged 60-69 years (45%), while moderate ED was also predominant in the same age group (25%). No cases of severe ED (0%) were reported in this age range, and a statistically significant correlation was found between increasing age and ED severity (p < 0.001). Duration of LUTS was another important factor, with mild ED most prevalent among patients reporting symptoms lasting for ≤12 months (87.0%). In contrast, moderate and severe ED were associated with longer symptom duration of up to 36 months. The mean duration of LUTS for patients with severe ED was 15.95 ± 12.44 months. LUTS severity was significantly associated with ED severity (p = 0.039). Patients with mild or moderate LUTS were more likely to exhibit mild ED (38.0% each), whereas severe LUTS was equally associated with mild and moderate ED (n = 22 each). Although PSA levels remained within normal limits across all categories of ED severity, a notable finding was the significant decline in testosterone levels among patients with more severe ED. Those with severe ED had a mean testosterone level of 285.94 ± 106.99 ng/dL, significantly lower compared to those with mild ED (p = 0.001), highlighting the impact of testosterone deficiency on ED severity (Table 2).

**Table 2:** Association of clinical histories of patients with different degrees of ED *Chi-square test, with significance at p < 0.05. **Analysis of variance (ANOVA). LUTS: lower urinary tract symptoms, IPSS: International Prostate Symptom Score, IIEF-15: International Index of Erectile Function-15, PSA: prostate-specific antigen.

Variables		Case (n = 183)	Erectile dysfunction (IIEF-15 score)			p-value
			Mild	Moderate	Severe	
			n = 92 (%)	n = 73 (%)	n = 18 (%)	
Age groups (years)	50-59	63	44 (47.8)	19 (26)	0 (0)	<0.001^*^
	60-69	70	45 (48.9)	25 (34.2)	0 (0)	
	70-79	39	3 (3.3)	28 (38.4)	8 (44.4)	
	>79	11	0 (0)	1 (1.4)	10 (55.6)	
Duration of symptoms (months)	≤ 12	151	80 (87)	57 (78.1)	14 (77.8)	0.056
	13-36	26	8 (8.7)	15 (20.5)	3 (16.7)	
	37-60	4	4 (4.3)	0 (0)	0 (0)	
	>60	2	0 (0)	1 (1.4)	1 (5.6)	
	Mean (± SD)	11.46 ± 10.73	11.07 ± 10.61	11.18 ± 10.13	15.95 ± 12.44	0.128**
LUTS (IPSS)	Mild	67	35 (38)	25 (34.2)	7 (38.9)	0.039^*^
	Moderate	62	35 (38)	26 (35.6)	1 (5.6)	
	Severe	54	22 (23.9)	22 (30.1)	10 (55.6)	
Serum PSA levels (ng/mL), mean (± SD)		1.86 ± 1.68	1.98 ± 2.18	1.74 ± 0.88	2.12 ± 1.13	0.862**
Testosterone levels (ng/dL), mean (± SD)		483.65 ± 155.26	544.09 ± 109.80	456.23 ± 166.72	285.94 ± 106.99	0.001^**^

Association between clinical histories of patients with different degrees of ED

The study explores the relationship between various clinical histories and the severity of ED in a cohort of 183 patients, categorizing ED into three levels (mild, moderate, and severe) based on the IIEF-15 score. A statistically significant association was observed between the presence of DM and ED severity in 36 patients (p = 0.027), showing a higher prevalence of moderate-to-severe ED. Hypertension (HTN) also has a significant correlation (p = 0.039), indicating that patients with HTN are more likely to experience moderate-to-severe ED. In contrast, coronary artery disease (CAD; p = 0.830) and other comorbidities (p = 0.451) have no significant relationships with ED severity (Table 3).

**Table 3:** Association of clinical histories of patients with different degrees of ED *Chi-square test, with significance at p < 0.05. HTN: hypertension, DM: diabetes mellitus, CAD: coronary artery disease.

Clinical history	Case (n=183)	Erectile dysfunction (IIEF-15 score)						p-value
		Mild		Moderate		Severe		
		n	Frequency (%)	n	Frequency (%)	n	Frequency (%)	
DM	36	25	27.2%	10	13.7%	1	5.6%	0.027^*^
HTN	38	26	28.3%	9	12.3%	3	16.7%	0.039^*^
CAD	14	7	7.6%	5	6.8%	2	11.1%	0.830
Other comorbidities	12	6	6.5%	6	8.2%	0	0%	0.451

Correlation between serum markers and the severity of ED

**Figure 1:**
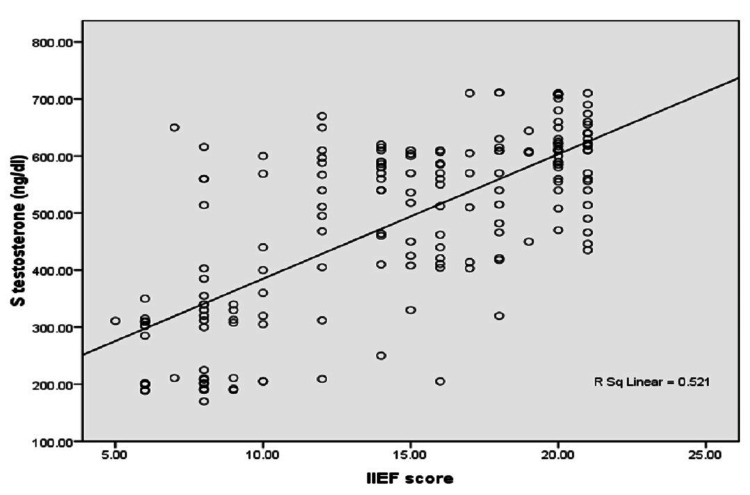
Correlation of serum PSA and testosterone levels with different degrees of ED PSA: prostate-specific antigen, ED: erectile dysfunction.

Testosterone levels exhibit a strong positive correlation with ED severity (Spearman's rho = 0.679, p < 0.001), indicating that lower testosterone levels are associated with more severe ED. In contrast, serum PSA levels show a negligible correlation with ED severity (Spearman's rho = 0.004, p = 0.962), suggesting no significant relationship (Table 4). Figure 1 represents these correlations, highlighting the significant association of testosterone levels with ED severity while showing no notable correlation for serum PSA.

**Table 4:** Correlation of serum PSA and testosterone levels with ED PSA: serum prostate-specific antigen, ED: erectile dysfunction.

Serum markers	Spearman's rho correlation coefficient	p-value
Testosterone levels (ng/dL)	0.679	<0.001
Serum PSA levels (ng/mL)	0.004	0.962

## Discussion

Our study highlights a significant correlation between LUTS and the severity of ED among elderly men (≥60 years). This association is consistent with global observations, underscoring the increasing prevalence of both conditions in the aging population. Studies have reported that the prevalence of LUTS increases with age, affecting up to 80% of men over 70 years, while ED is estimated to affect 40%-70% of men aged 60-70 years, with even higher rates in older populations [17]. The unavailability of Indian data for comparison within a national context presents a notable limitation of this study. The data reveal that 38%-44% of men aged 70-79 years experienced moderate-to-severe ED, aligning with previous studies that report similar prevalence rates [16,18]. Nordin et al. also noted a prevalence of moderate-to-severe ED in Malaysia ranging from 40.8% to 46% [19]. Aging impacts several physiological systems, including the nervous, vascular, and hormonal systems, all critical for erectile function [19]. This contributes to the perception of ED as a common consequence of aging.

In this study, a high prevalence of ED was found among patients with LUTS, with approximately 70% of the cohort showing mild-to-moderate LUTS. The frequency of ED was notably higher among these patients, which is in line with studies showing a strong association between LUTS and ED. A multicenter study in Russia found that 48.9% of men had ED, with a higher frequency of mild ED (34.6%) and varying degrees of LUTS [20]. Similarly, Wang et al. reported that ED was present in 76.39% of patients with mild LUTS, 87.32% with moderate LUTS, and 95.24% with severe LUTS [21]. Despite these findings, the exact molecular and pathological mechanisms linking LUTS to ED remain unclear. Furthermore, medical treatments for LUTS/BPH could potentially influence erectile function, which warrants further investigation. Although we identified a significant association between ED and chronic conditions such as DM (p = 0.027) and HTN (p = 0.039), this is consistent with existing literature, which recognizes HTN and diabetes as independent risk factors for ED [22]. Previous research has also highlighted an increased prevalence of ED in patients with cardiovascular disease [23].

The mean serum PSA levels in our study were within the normal range (2.12 ± 1.13 ng/mL for severe ED), and no significant association was observed between PSA levels and ED [24]. This finding aligns with previous studies, which reported similar median PSA values and found no strong correlation between PSA levels and ED [25]. In this study, no Indian data were available to determine whether there are differences in PSA levels, highlighting the gap in regional comparative studies. PSA is commonly used as a marker for prostate volume and LUTS severity, but its role in ED remains less clear. A significant positive correlation was observed between the IIEF-15 score and serum testosterone levels (r = 0.679, p < 0.001), highlighting the critical role of testosterone in erectile function. Our study found that patients with severe ED had notably lower testosterone levels compared to those with mild ED (285.9 ± 106.9 ng/dL vs. 544 ± 109.8 ng/dL). These findings are consistent with previous research indicating that low testosterone levels are associated with severe ED [26,27]. A meta-analysis also supports the link between low testosterone and ED, with about one-third of men with ED exhibiting testosterone deficiency [28]. This suggests that addressing testosterone deficiency may have clinical benefits for patients with ED. In this study, no Indian data were available to define the optimal testosterone levels in ED patients, which limits the applicability of our findings to the Indian population.

Limitations

A cross-sectional design of the study precludes making causal inferences about the associations between ED and different variables. This study's exclusive focus on a single hospital restricts the applicability of the results to larger populations. Moreover, although the study examines testosterone levels, it does not investigate other important hormonal or biochemical indicators that could contribute to a more thorough comprehension of erectile dysfunction. The aforementioned constraints indicate the necessity for longitudinal, multicenter investigations with larger samples and more comprehensive biomarker analysis to validate and expand upon these results.

## Conclusions

This study reveals a strong correlation between ED and the severity of LUTS in men with BPH, underscoring the interrelated nature of these conditions in the aging male population. It also shows that increasing age, low testosterone levels, and the presence of comorbidities such as HTN and DM are significant risk factors for ED in men with BPH. Comprehensive evaluation and management strategies for LUTS and ED should consider these factors to improve patient outcomes. Further research is required to investigate the underlying mechanisms connecting BPH, LUTS, and ED for the targeted interventions. The absence of Indian-specific data in this study highlights a critical gap, emphasizing the need for future studies to establish context-specific clinical practice in India.
